# Shifts in bird ranges and conservation priorities in China under climate change

**DOI:** 10.1371/journal.pone.0240225

**Published:** 2020-10-08

**Authors:** Ruocheng Hu, Yiyun Gu, Mei Luo, Zhi Lu, Ming Wei, Jia Zhong

**Affiliations:** 1 Center for Nature and Society, School of Life Sciences, Peking University, Beijing, China; 2 Shan Shui Conservation Center, Beijing, China; 3 China Birdwatching Association, Yunnan, Kunming, China; Sikkim University, INDIA

## Abstract

Climate change is one of the most significant causes of species range shift and extinction. Based on a citizen science dataset of birds in China, the Bird Report, we developed a high-resolution map of bird species richness in China, and simulated the range shifts and area changes of the 1,042 birds through the year 2070 using three different General Circulation Models and two different Representative Concentration Pathways (RCPs, including RCP 2.6 and RCP 8.5). It was found that 241–244 (under different scenarios) bird species would lose a portion of their distribution ranges; and that most species in China would move to either higher elevations or northward. The other 798–801 species would experience range expansion. Compared to resident species (n = 516), migratory birds (n = 526) may undergo more limited range expansion but a longer range shift distance on average. The species diversity of birds will considerably increase in areas higher than 1,500 m in elevation under both RCPs. Conservation priorities with higher species richness were also identified using the Zonation model. The existing national nature reserves are not sufficient for protecting important bird habitats, especially after range shifts. Significant gaps in protected areas were observed in the northern Xinjiang, southern Tibet, Greater Khingan, Sanjiang Plain, Songnen Plain, northern Bohai Rim, and southeastern coastline areas. Many of these areas are characterized by high human populations and intensive development, and establishing sizable protected areas has become difficult. Inclusive conservation mechanisms that include restoring habitats in urban parks and sharing habitats in farmland areas, may be a feasible solution.

## 1. Introduction

Biodiversity conservation must take the impact of climate change into account. By 2100, 68% of terrestrial and 39% of tropical marine assemblages are predicted to have more than 20% of their constituent species exposed to unprecedented temperatures [1]. Growing evidence shows that climate change can cause geographic range changes including range area, latitude, and elevation shifts [2–7] in many species, such as lizards, corals, and birds [8–10], which may increase species extinction risks [11–13]. In the context of conservation management, the range shift of each species may lead to a change in conservation priority areas [13]. On a world scale, studies have presented maps of changes in species distribution ranges (birds, amphibians, and mammals) under future climate change scenarios [14, 15], and noted that biodiversity hotspots may move to high-latitudes. However, these world-scale studies have low spatial resolution and use polygon range data based on expert experience, that may lead to worse estimates of underlying environmental variables [16, 17]. Therefore, these results are difficult to use to guide the planning and management of protected areas in response to climate change at the country scale. Notably, regional or national-scale studies are still necessary because different countries have different species structures.

In Europe, a study predicted that 431 breeding bird species may remain in only 72–89% of the current distribution range by 2099 [2]. The United Kingdom and Australia have also published national assessment reports on the biodiversity responses of species to climate change [18, 19]. However, China is one of 17 mega-diverse countries in the world, and the National Action Plan on Climate Change of China 2014–2020 did not mention the future climate impact on biodiversity [20].

In China, impacts of future climate change on the distribution of species have only been studied for a few species groups. Regionally, on the Tibetan Plateau, ungulates may experience 30%-50% range loss and 300-km poleward shifts on average by 2080 [21]. At the national scale, before the next century, 135 endemic or endangered species, including amphibians, reptiles, and mammals, may experience 50% habitat loss [22]. A study of 44 migratory waterbird species found that the conservation hotspots of these species would move northward by 2050 [23]. Moreover, the data sources and simulation methods used in the above studies are not uniform, making comparison difficult, and attention should not only paid to threatened and endemic species in order to understand the shifts of diversity and richness patterns [24]. Although birds are an important ecological indicator of climate [25], an overview of the full taxon of avian species in China has not been obtained.

With limited budgets and various extinction risks for different species, planning an active conservation network in response to future climate change scenarios remains a major challenge [26, 27]. To make planning more effective, convenient but powerful models based on multispecies distribution range data have been developed. For example, Pouzols et al. used the Zonation model to map the details of global protected areas needed by 2040 [28], and Levy and Ban used the Marxan model to incorporate climate change modeling into marine conservation [29]. Therefore, it is feasible to develop rigorous assessments and conservation plans, based on species range data.

Range maps downloaded from the databases of IUCN Red List and Birdlife International are a widely used data sources for biogeography research [30]. However, these range maps, based on the expert knowledge, may have more biases than maps based on occurrence data from species distribution models [16]. For conservation planning, citizen science has played an essential role to produce data with accuracy equal to or surpassing that of experts if the data is carefully verified and applied [31–34]. The occurrence data with the time sequence from citizen science projects have filled the gaps in continuous data to analyze the effects of climate change on species distributions, such as the changing migration patterns of birds in North America [35]. In China, where historical occurrence data are scarce and difficult to digitize [36], citizen science methods such as birdwatching have provided much more occurrence data than scientific papers, including for threatened and rare species [37]. These citizen science datasets have been widely and independently used in national-scale studies of China to explain issues such as the effects of climate change on biogeography and conservation planning for both individual species and multiple species [23, 36, 38, 39]. Although such data may show observation bias, reporting bias, and geographic bias due to lack of a standardized field protocol [40], these useful large-scale and full-taxon data can be used with data cleaning and correction [41]. In fact, for modeling range shifts under climate change scenarios, occurrence data from birdwatching is the only choice in China.

In this paper, we collected 161,630 localities of 1,111 bird species in China from Bird Report as occurrence data and applied the dataset to MaxEnt [42] to predict distribution range maps of each species both at present and in 2070. We also mapped the conservation priorities of birds in China and the corresponding shifts in different climate scenarios using the Zonation planning model based on these maps and the percentage change of each species range. We aim to understand the pattern and direction of each species range shift, as well as variations in bird hotspots, to provide insights for future conservation priorities.

## 2. Material and methods

### 2.1 Data sources

We collected occurrence (presence-only) data for birds from a citizen science project, Bird Report (http://www.birdreport.cn/). Bird Report is the largest nationwide project involving the submission of birdwatching records in China, including records of more than 1,390 species of birds (approximately 92% of bird species in China) since 1998 [36]. To ensure the accuracy of location and species identification information, each submitted record was checked by an experienced reviewer (only birdwatchers who have submitted more than 300 bird species and 100 birding reports in China are eligible to become reviewers). After collecting 4.7*10^4^ birding reports (each report may contain dozens of records) from 1998 to 2017, we verified the accuracy of coordinates from the place names of each bird record according to the Google Maps API. We used all occurrence data (including breeding records and migratory records) for a single species because different subpopulations of many species may have different reproductive and migratory habits at such a large scale, making it difficult to clearly distinguish between breeding and nonbreeding occurrence data from solo birdwatching records.

Because bird watching does not always meet the requirements of systematic sampling [43], we measured the uniformity of the dataset for all localities by using the Measuring Geographic Distributions toolset in ArcMap 10.2 (ESRI 2010, Redland, California). We found that 68% of the occurrence data are distributed in 44% of the country's land area (by calculating the standard distance concentrated circle), and the distance between the median center and the mean center of features was 226.2 kilometers. This result suggested that there is indeed a bias, which may be due to gaps in species richness, the population density, and the traffic status in different regions. To avoid overfitting and high -variability predictions, we used two criteria: 1) bird species with fewer than five independent localities were excluded [44]; 2) if localities are highly concentrated for a species (the distance between any two localities is less than one arc-minute), the excess localities were randomly removed (presence thinning). This data cleaning process has been proven to effectively match the results of the MaxEnt model and improve model performance [36, 39, 45–47]. Finally, we used 161,004 independent localities to create modeled distribution range maps for 1,042 bird species (for details, see S1 Table).

We used all the localities collected for each species to model the total distribution range of that species. We did not determine whether these localities were breeding or nonbreeding sites. Because eastern China is an important part of the East Asian—Australasian Flyway (EAAF), many species such as geese and shorebirds breed in northern China or Russia, migrate past eastern China [48], or winter in southern China [49]. If only breeding ranges are considered, the key resting and wintering habitats for these birds to survive may be omitted from the conservation plan, and these potentially missing habitats may face serious threats [39, 50, 51]. As a reference, we distinguished the localities and mapped the breeding ranges of migratory bird species in China for comparison with other similar research results (for details, see S1 File & S2 Table).

To forecast the response of species distribution to future climate change, it is necessary to select appropriate variables to construct species climate niches by considering the complex interactions among bioclimatic variables and digital elevation model (DEM) data [52–55]. To avoid overfitting caused by the autocorrelation between variables (especially in projections of future scenarios), we calculated the Pearson’s correlation at all presence sites for every pairwise combination of 19 bioclimatic variables downloaded from the WorldClim 1.4 database [56] (ESRI grids; bio 30s). Among all pairwise combinations, the variable with the most "high correlation pairs" (|r| > 0.9) was excluded [57]. Next, we selected the remaining 12 bioclimatic variables for modeling: bio 1–5 (Annual Mean Temperature, Mean Diurnal Range, Isothermality, Temperature Seasonality, and Max Temperature of Warmest Month), bio 8–9 (Mean Temperature of Wettest Quarter and Mean Temperature of Driest Quarter), bio 12 (Annual Precipitation), bio 14–15 (Precipitation of Driest Month and Precipitation Seasonality), and bio 18–19 (Precipitation of Warmest Quarter and Precipitation of Coldest Quarter). To predict the ranges of birds in the future, we used three different General Circulation Models (GCM) for 2070 based on CMIP5 data, in both RCP 2.6 and RCP 8.5 scenarios: CCSM4, HadGEM2-ES, and MIROC5. These GCMs have been proven to perform well for vertebrates [6, 10, 21, 22]. We use the RCP2.6 and RCP 8.5 scenarios because they represent the lowest and highest level of emissions respectively. From this approach, we could derive the lower and upper limits of the impact on the bird species distribution [58]. All bioclimatic variables of these GCMs were downloaded from WorldClim 1.4. Data for National Nature Reserves (NNRs) in China were collected from existing research [36]. All grid data were rescaled to 30 arc-seconds (~1 kilometer) in ArcMap 10.2.

### 2.2 Distribution range modelling

We used MaxEnt 3.4.1 to model range maps [42]; this software provides robust results by implementing a machine learning approach for presence-only data (such as citizen science data) [59, 60]. The model established based on cross-validation by five replicates (each replicate used 80% of the data for training and 20% of the data for test) for each bird with Cloglog output. Cloglog transformation is an updated algorithm in MaxEnt 3.4.1 for estimating the probability of presence by yielding a Bernoulli generalized linear model based on the Poisson distribution [61]. Compared with the traditional Logistic transform used in previous MaxEnt versions, Cloglog transformation can improve model performance to reduce the effects of sample selection bias while maintaining the same area under the receiver operating characteristic curve (AUC) value [42]. The background in MaxEnt was set as the entire land area of China for all species. After modeling the distribution range in the current scenario, we projected the model with different GCMs for RCP 2.6 and RCP 8.5 in 2070. We wrote R scripts to automatically batch the modeling process (for the code, see S2 File).

The average Cloglog output for each bird and scenario was used to produce the species range map. The maximum test sensitivity plus specificity (MTSS) values for each species were used as thresholds to convert Cloglog raster outputs to “presence/absence” binary maps [62, 63], named predicted distribution range maps (DRMs).

In order to improve the performance of the model, we conducted two rounds of simulation. In the first round, all 1,111 species with 161,630 localities and all 13 environment variables in current scenario (12 bioclimatic variables and the DEM) were applied to MaxEnt model by five cross-validated replicates with no special settings. We analyzed the results of first round process, collected the area of each DRM and the contribution value of each variable for each species. We excluded 69 species with few localities’ density by the criteria that the number of points less than 1 per 3° * 3° (about 1 * 10^5^ km^2^) in the DRMs, and excluded variables that had no contribution in the model for each species [64]. Finally, we used 1,042 species with 161,004 localities for second round simulation.

In the second round, we used different environment variables for each species (details see S1 Table). Background selection method is also used for each species for sampling bias correction, which had been proven useful in data-sparse situations [46, 65]. We collected province-level distribution records of each species [66] as the background selection sampling in the MaxEnt, aiming to create statistically reliable results [67]. Other settings are the same as the first round. The code for background selection and variables selection shown in S2 File. The mean training AUC value for 1,042 species was 0.908±0.055, and the mean test AUC value was 0.861±0.085 (see S1 Table).

We overlaid all DRMs from the second-round simulation of 1,042 species to produce species richness maps for the current scenario and the 2070 scenario. We projected these richness maps on the DEM as a 3D model by ArcMap to show the change related to the terrain. To measure the effect of climate change on the shift direction of the distribution range, we identified the median center point (the location that minimizes the overall Euclidean distance to the maps) of each range map under different RCPs. Then, we calculated the displacement vectors of the distribution range under different scenarios.

### 2.3 Conservation priority modeling

We used Zonation v4.0 as the planning tool to generate conservation priority maps for different RCPs [28]. Zonation is capable of large-scale, high-resolution spatial conservation prioritization with big data, and it can achieve a higher conservation value aggregated across biodiversity features and space than other planning software [68]. All original Cloglog output files of species for each RCP scenario were applied as species layers in the tool to retain information for the distribution maps. We used the percentage change value of the distribution range of each species as the weight value in Zonation to indicate the impact of climate change (species with high habitat loss were given high weight values, and species with high habitat increases were given low weight values). We added an extra 0.5 to the weight value if the species is endemic to China (for details, see S1 Table). Considering the conservation value of each species, we used the basic core-area zonation (CAZ) result for cell removal rule and set the wrap factor to 1,000; thus, we were able to identify areas with high occurrence levels for a single rare/highly weighted species [69].

We produced two model scenarios for each RCP to meet different conservation planning target: 1) Random ranked scenario that only considering species layers and their weights; 2) NNRs masked scenario that aim to map priorities out of NNR regions by apply the additional NNR layer as the mask layer in the Zonation.

## 3. Results

### 3.1 Identifying the species with the range reduction and expansion

**Fig 1:**
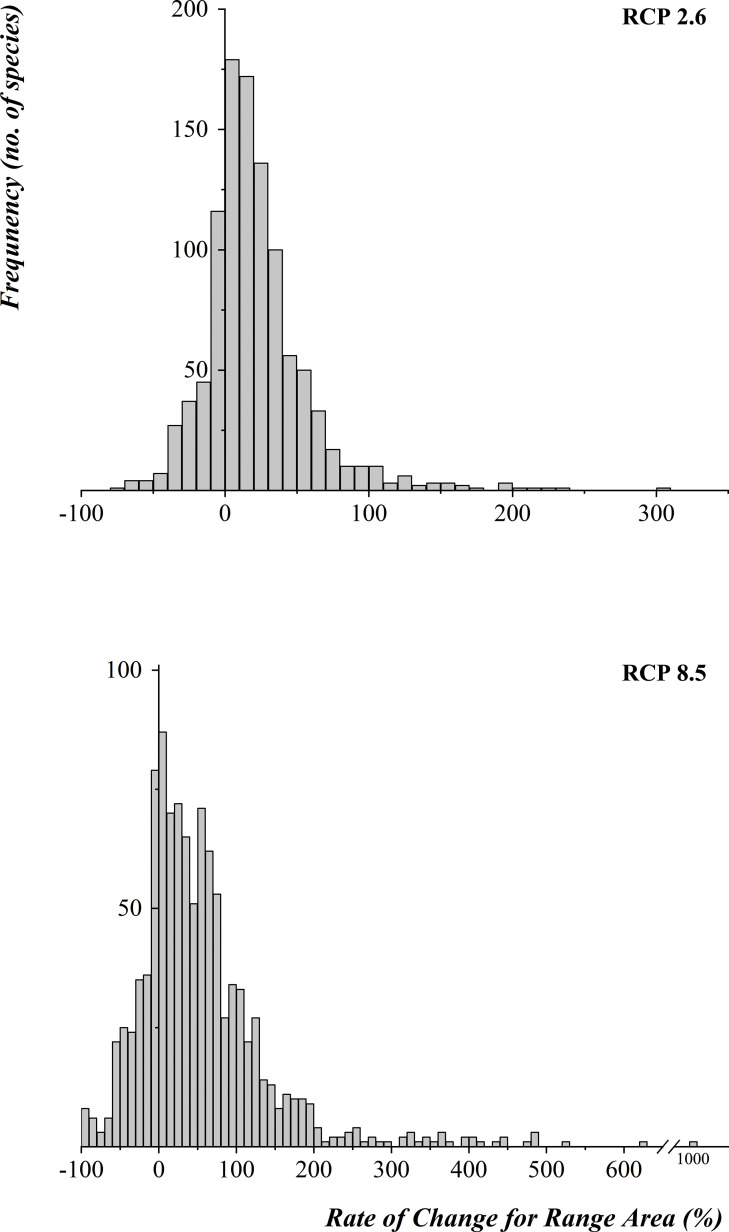
Histograms of distribution range change for bird species in 2070.

Among the 1,042 studied species, approximately 25% of bird species may lose part of their range, and approximately 75% of may expand their distribution range due to the climate change by 2070 (Table 1) under RCP 2.6 or RCP 8.5. The histogram of range change for all bird species (Fig 1) shows that in general, species will have a broader range under RCP 8.5 than under RCP 2.6 (Z = -4.49, P-value = 3.5E-6), although more range may be lost under RCP 8.5 for the 30% species that reduce their ranges (Table 1). For RCP 2.6, we found that 30 species with reduced ranges are threatened birds, with an average of 19.41% range loss. In the meantime, 31 threatened species may experience an average range expansion of 39.24%. For RCP 8.5, we found that 30 threatened species are projected to experience an average range loss of 33.55% (nine of them would lose more than half of their range), and 31 threatened species would potentially experience an average range expansion of 98.89%. We also found that climate change has more significant effects on the range changes of threatened species than other species, whether for RCP 2.6 (P-value = 0.019) or RCP 8.5 (P-value = 0.046). We divided the studied species into migratory (*n* = 526) and resident (*n* = 516) birds, and counted their range changes separately (Table 1). The statistics showed that the impact of climate change on the range of resident species is more intense than that for migratory species, whether under the RCP 2.6 scenario (P-value = 0.0001) or the RCP 8.5 scenario (P-value = 0.026), indicating that the ranges of resident species will experience relatively broad range expansion. For the 190 sample breeding species, the breeding range may change less than the total distribution range, regardless of the climate scenarios (P-value = 0.005 for RCP 2.6, P-value = 0.003 for RCP 8.5; for details, see S1 Fig).

**Table 1:** Statistics of range area changes for bird species in different climate change scenarios.

Group Scenario	All (*n* = 1,042)		Migratory (*n* = 526)		Resident (*n* = 516)	
	Range reduce	Range expand	Range reduce	Range expand	Range reduce	Range expand
RCP 2.6	*n* = 241	*n* = 801	*n* = 149	*n* = 377	*n* = 92	*n* = 424
	*r* = -15.7	*r* = 32.6	*r* = -16.3	*r* = 30.4	*r* = -14.8	*r* = 34.7
RCP 8.5	*n* = 244	*n* = 798	*n* = 139	*n* = 387	*n* = 105	*n* = 411
	*r* = -27.5	*r* = 79.8	*r* = 30.2	*r* = 76.7	*r* = -23.8	*r* = 82.7

*n*: No. of species; *r*: Average percentage change in the range.

We listed the top ten species with the largest range changes, as shown in S2 Fig. More than half of the species with reduced ranges are migratory birds (60% of the species in both RCPs). It is worth noting that many of them are threatened or endemic to China; notably, Przevalski's Rosefinch (*Urocynchramus pylzowi*), Taiwan Blue Magpie (*Urocissa caerulea*) are endemic species of China; the Red-crowned Crane (*Grus japonensis*) is endangered bird species. All of these species would lose more than half of their distribution ranges under RCP 2.6 or more than 80% of their range areas under RCP 8.5. Among them, Short-tailed Parrotbill (*Paradoxornis davidianus*) may go extinct in China (may lose 99.98% range under RCP 8.5). We found that none of the ten species with expanded ranges are threatened or endemic, and all of these species are mainly distributed in southern and southwestern China (see S2 Fig). The distribution ranges of these species were projected to be at least doubled (RCP 2.6) or quadrupled (RCP 8.5). Notably, the range of the Pied Falconet (*Microhierax melanoleucus*) was projected to increase ten-fold under the RCP8.5 scenario.

### 3.2 Multidimensional shifts in bird ranges

The statistics for the range shifts of different species groups show that under climate change in 2070, more species would move to the north than to the south (P-value = 0.034 for RCP 2.6, P-value = 0.031 for RCP 8.5), with longer average shift distances (P-value = 0.008 for RCP 2.6, P-value = 0.004 for RCP 8.5) across different species groups (Table 2). This result shows that the northward shift of the distribution range may be a holistic trend.

**Table 2:** Statistics for the range shifts of bird species under different climate change scenarios.

Group Scenario	All (*n* = 1,042)		Migratory (*n* = 526)		Resident (*n* = 516)	
	To the North	To the South	To the North	To the South	To the North	To the South
RCP 2.6	*n* = 831	*n* = 211	*n* = 459	*n* = 67	*n* = 372	*n* = 144
	*l* = 103.5	*l* = 43.5	*l* = 110.6	*l* = 35.3	*l* = 94.8	*l* = 47.3
RCP 8.5	*n* = 819	*n* = 223	*n* = 440	*n* = 86	*n* = 379	*n* = 137
	*l* = 187.0	*l* = 87.2	*l* = 202.5	*l* = 89.5	*l* = 169.1	*l* = 85.7

*n*: No. of species; *l*: Average shift distance (kilometers).

**Fig 2:**
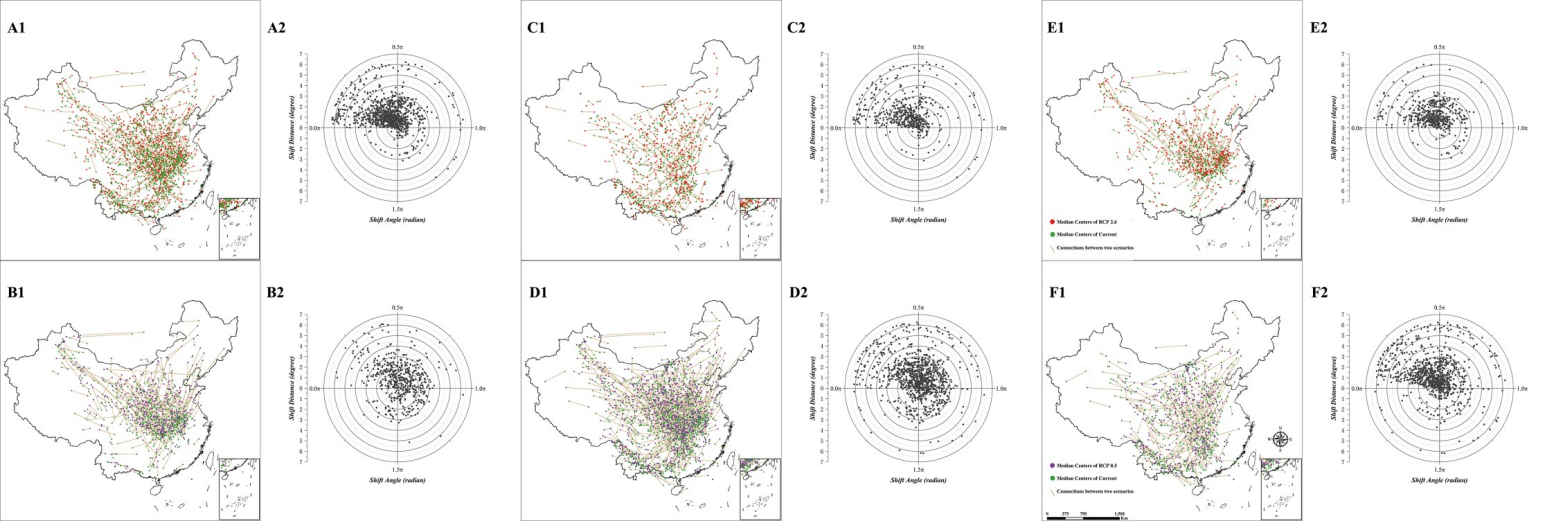
Range shifts of bird species in 2070. The first line and the second line show the patterns under RCP 2.6 and RCP 8.5, respectively. The three sets of charts arranged in columns show the “all species” group (A-B), “migratory” group (C-D), and “resident” group (E-F) separately. The boundaries are reprinted from Shan Shui Conservation Center under a CC BY 4.0 license, with permission from Xiangying Shi, original copyright 2020.

The displacement vectors of each bird species range are mapped over the geographical scope of China and represented in polar form to understand the detailed spatial patterns of range shifts under both the RCP 2.6 and RCP 8.5 scenarios in 2070. For RCP 2.6, the birds distributed in North Xinjiang, North China, and Northeast China would move northwards, and those in South Qinghai would move to the Tibetan Plateau mountains in the south (Fig 2A1). After subdividing the direction of movement, we found that approximately more than half of the species (547/1042) would move to the northeast with long average shift distances, and a quarter of species would move to the northwest (284/1042), with short average distances. For the remaining quarter of the species, 135 species would move to the southwest, and the other 76 species would move to the southeast, both having short average shift distances (Fig 2A2). Whether the migratory group (Fig 2C1 and 2C2) or resident group (Fig 2E1 and 2E2), the number of species in the four quadrants of the polar chart is very similar to the pattern exhibited by all species (Fig 2A2), with no significant differences (P-value = 0.34 for the migratory species & all species pairs, P-value = 0.30 for the resident & all species pair).

For RCP 8.5, the basic pattern of range shifts remains the same but the migration distances are longer and more variable than those in the previous scenario (Fig 2B1). Compared to those in the RCP 2.6 scenario, the directions of range shifts are more concentrated to the northeast (54.8% of species) in this scenario, with a longer average distance (Fig 2B2). For the migratory group (Fig 2D1 and 2D2), the proportion of species moving to the northeast decrease from 25.7% to 21.3%; for the resident group (Fig 2F1 and 2F2), there would be no significant change in the shift direction pattern, but the shift distances of species may be longer. It is worth noting that 50 species are predicted to move to the north under RCP 2.6 but to the south under RCP 8.5, i.e., in the opposite direction; and 38 species may move to the south under RCP 2.6 but move to the north under RCP 8.5.

**Fig 3:**
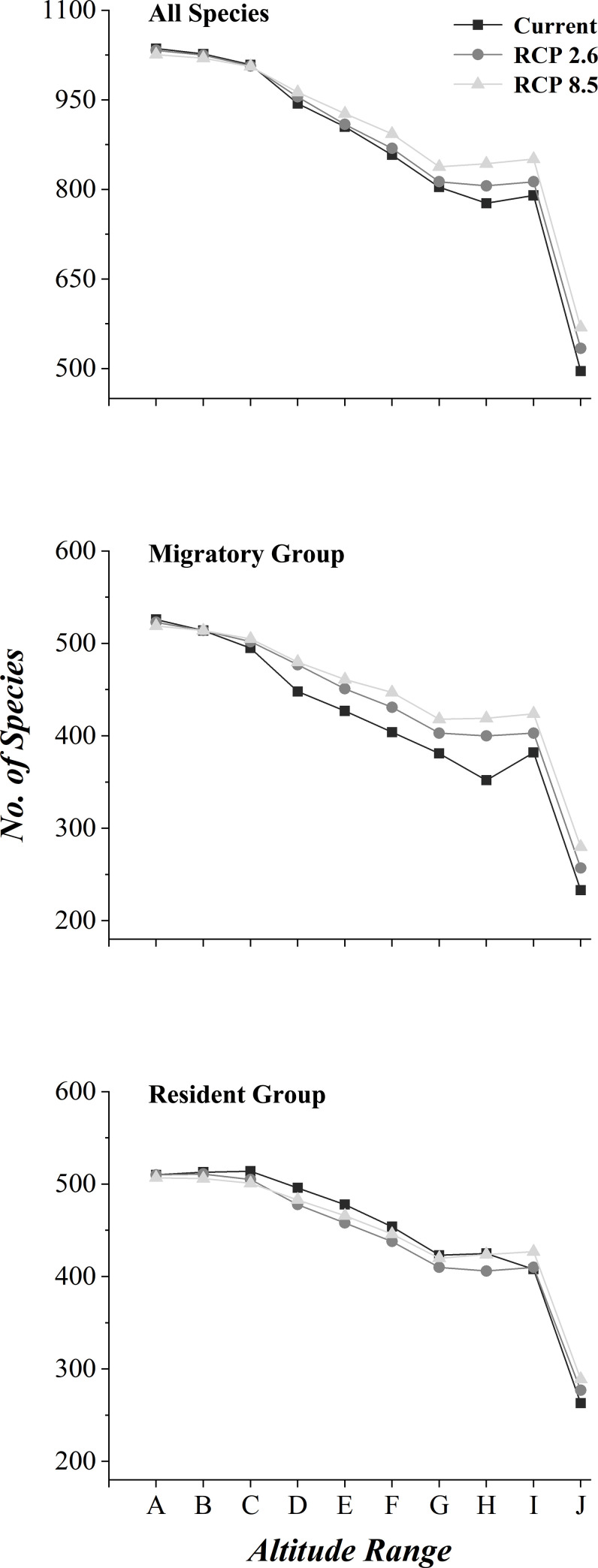
Species diversity at different elevation ranges in different climate change scenarios. Elevation: A (0–500 m), B (500–1,000 m), C (1,000–1,500 m), D (1,500–2,000 m), E (2,000–2,500 m), F (2,500–3,000 m), G (3,000–3,500 m), H (3,500–4,000 m), I (4,000–5,000 m), J (>5,000 m).

In both scenarios, most bird species would move to higher elevations by 2070 (Fig 3). Therefore, the species diversity would increase in mountains and plateaus and decrease in lower elevations. We measured species diversity changes in elevation and found that, if put all species studied together, the turning point would be 1,500 meters in elevation, with the diversity increase above it and decrease below it. Further analysis revealed that, this turning point would be much lower for migratory species, 500 meters in elevation, and much higher for resident species, 4,000 meters in elevation. This probably suggested that more resident birds may lose their lower elevation habitats.

### 3.3 Richness pattern change for bird species

**Fig 4:**
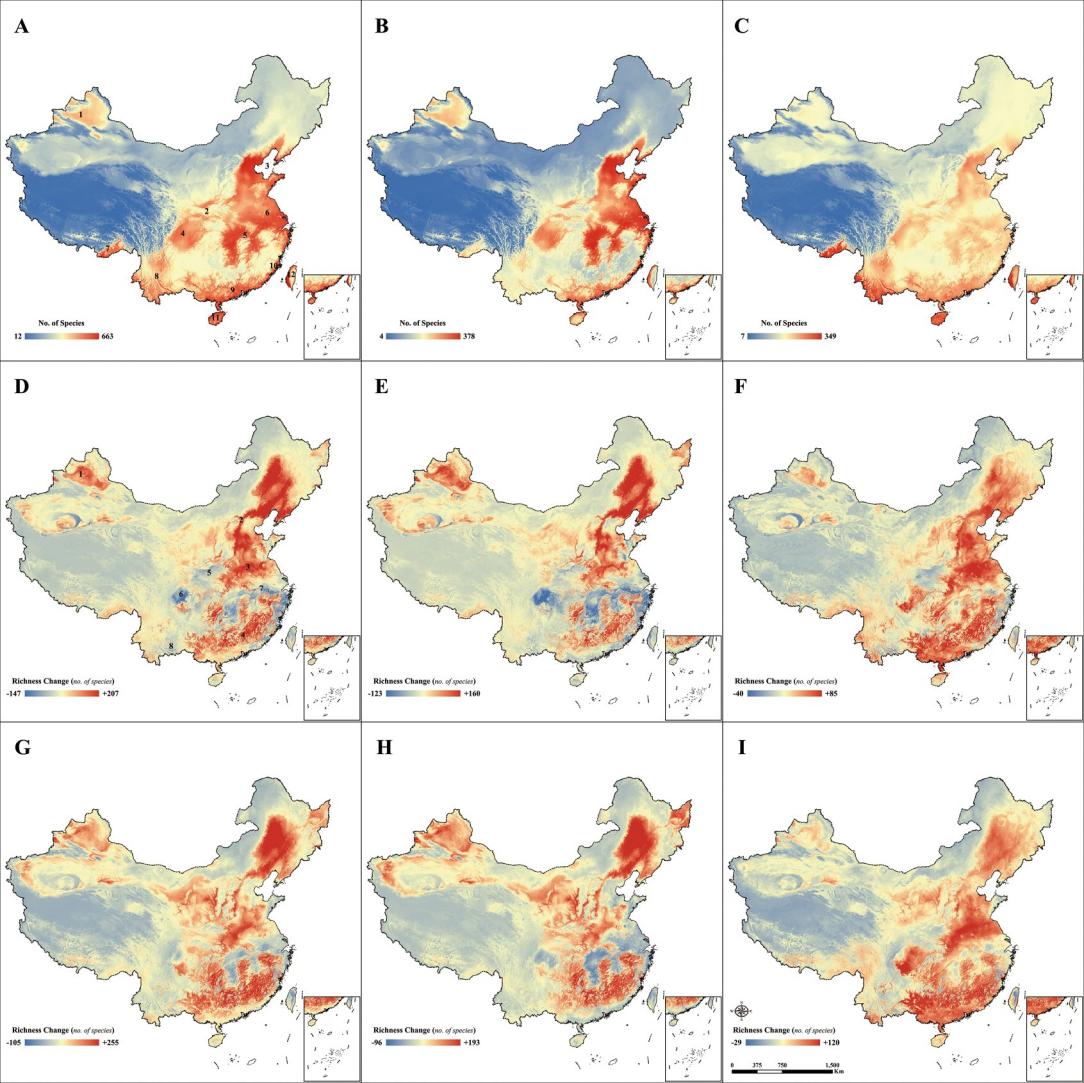
Richness maps and richness changes under different climate change scenarios for bird species in China. The figures arranged in three columns show “all species” group, “migratory” group, and “resident” group separately. A-C are the current richness maps; D-F are the richness changes under RCP 2.6; and G-I are the richness changes under RCP 8.5. The boundaries are reprinted from Shan Shui Conservation Center under a CC BY 4.0 license, with permission from Xiangying Shi, original copyright 2020.

By overlaying the DRMs of the current scenario, we developed the first high-resolution full-taxon species richness map of birds in China (Fig 4A). The map shows that regions with high species richness include 1) northwestern Xinjiang, 2) the Qinling-Daba Mountains, 3) the Bohai Rim region (the North China Plain, the Liaodong Peninsula and the Shandong Peninsula), 4) the Southwestern Mountains and western Sichuan Basin, 5) the Hunan and Jiangxi Plains, 6) the lower Huai River & Yangtze River Basin, 7) southeastern Tibet, 8) central and south Yunnan, 9) the Lingnan Area, 10) southeastern coastal areas, 11) the Hainan Island, and 12) western Taiwan Island. The migratory bird species were mainly distributed in regions such as the region 1–6, region 9, 10, 12 (Fig 4B). The high species richness regions of the resident species include regions 7–9, 11 and 12 (Fig 4C).

After incorporating climate scenarios into the model, we found that the richness pattern of bird species in China changed significantly (Fig 4D–4I). Overall, bird species richness will increase in North and East China (especially in plain and mountain areas) and decrease in South and West China (especially in river areas and lowlands) under both RCP 2.6 and RCP 8.5 scenarios (original richness maps considering climate change are shown in S3 Fig).

For the RCP 2.6 scenario, we predicted that areas with an increase in the richness of all studied species would be in 1) northern Xinjiang, 2) the North & Northeast China Plain, 3) the Huai River Basin, and 4) the Southeast Hills. Areas with species richness decreases may include 5) the Han River Basin, 6) the Sichuan Basin, 7) the Lower Yangtze Plain, and 8) southeast Yunnan (Fig 4D). The pattern of richness change for migratory species is similar to that shown in Fig 4D, except for the difference in the southwestern region, which is not apparent (Fig 4E). For resident species, most of the regions would experience a richness increase, excluding regions 5, 7 and 8 (Fig 4F). The pattern of richness change for the three species groups in RCP 8.5 was similar to that in RCP 2.6, and the richness growth in Xinjiang was significant (Fig 4G–4I). We also found that regardless of the scenario, the richness change for resident species might be smaller than that for migratory species. If we do not consider wintering and migratory ranges, then we would lose nearly a quarter of the species (*n* = 226) that could be analyzed in the richness pattern map (S1 Fig), and we will ignore high-species-diversity-regions 5, 6, 8, and 9–12 in Fig 4A.

Based on the 3D scene model (S1 Video), we found that the species richness of birds in high-elevation areas would increase in both emission scenarios, and the change might be even more apparent when total emission increase (from RCP 2.6 to RCP 8.5). This phenomenon is most pronounced in rugged areas with significant elevation changes, such as the mountain valleys in southern Yunnan, the Brahmaputra-Himalayas Intersection in southeastern Tibet, the northwest edge of the Sichuan Basin, the southern edge of the Hengduan Mountains, the eastern side of the Tibetan Plateau, and the northwestern edge of the Tianshan Mountains.

### 3.4 Conservation gaps for future priority areas

**Fig 5:**
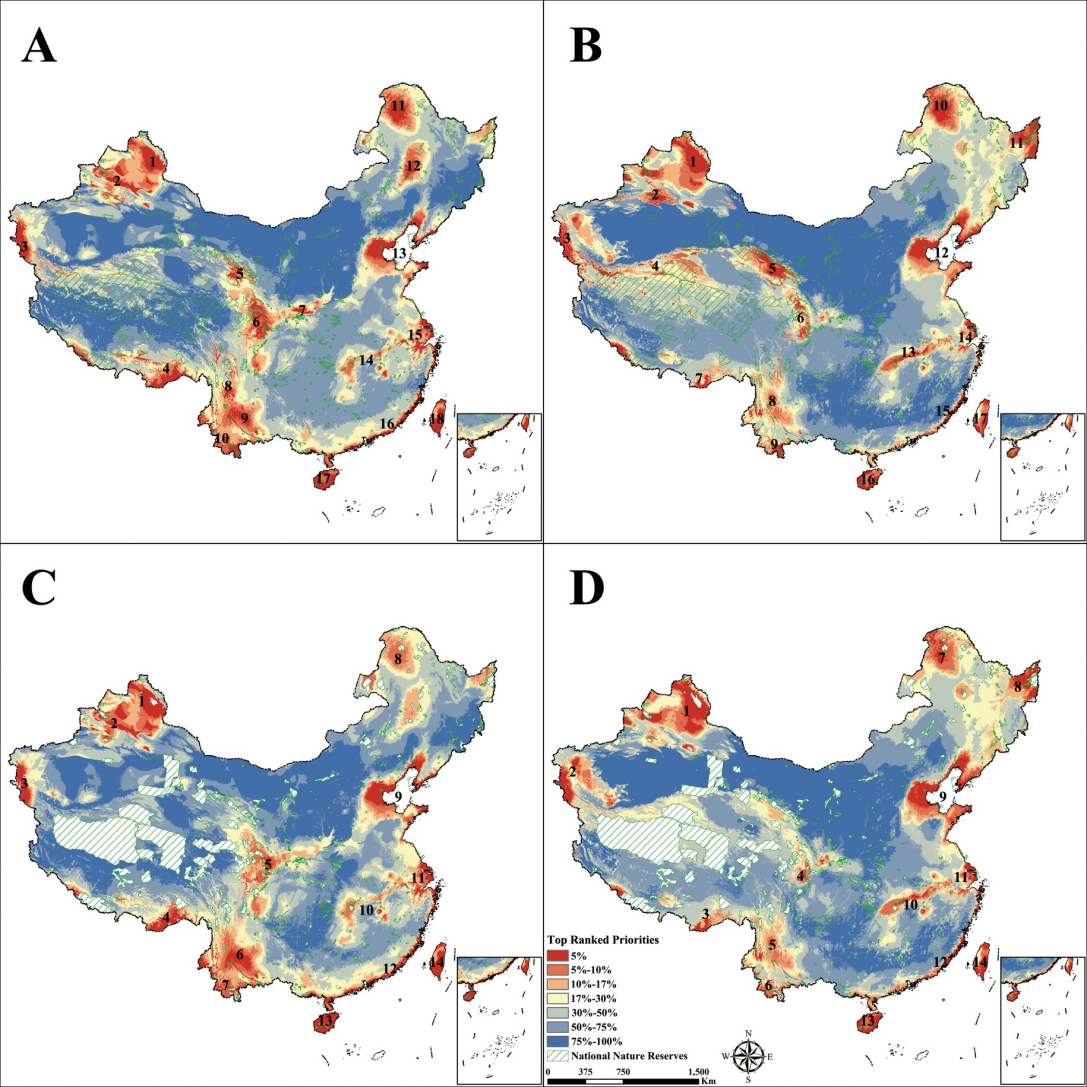
Ranked conservation priority maps for bird species in 2070. A and C show the priorities under RCP 2.6, B and D show priorities under RCP 8.5; A and B show the random ranked scenario, C and D show the NNRs masked scenario. The boundaries are reprinted from Shan Shui Conservation Center under a CC BY 4.0 license, with permission from Xiangying Shi, original copyright 2020.

Priority maps of birds based on range areas and the percentage changes for distribution range show the priority candidates for new protected areas (Fig 5; A and B show the random scenario, C and D show the NNRs masked scenario). The updated Zero Draft of post 2020 CBD Framework proposed a new target of 30% of territorial areas for biodiversity conservation [70], which we used to obtain the ranges of priority areas (shown in red, orange and yellow in of Fig 5).

For RCP 2.6 in random scenario (Fig 5A), the top priorities included the 1) the Altai Mountains; 2) the Tianshan Mountains; 3) the Karakoram Mountains; 4) the southeastern Tibet basin; 5) the eastern Qilian Mountains; 6) the three-province border area of Qinghai, Gansu, and Sichuan; 7) the Qinling Mountains; 8) the Three Parallel Rivers areas; 9) central Yunnan mountains; 10) south Yunnan; 11) the Greater Khingan area; 12) the Songnen Plain; 13) the Bohai Rim; 14) the central Yangtze River Basin; 15) the Yangtze River Delta and the Hangzhou Bay; 16) southeastern coastline areas; 17) the Hainan Island; and 18) the Taiwan Island.

For RCP 8.5 (Fig 5B), the top priorities include 1) the Altai Mountains; 2) the Tianshan Mountains; 3) the Karakoram Mountains; 4) the western Kuenlun Mountains; 5) the Qilian Mountains; 6) eastern Sanjiangyuan area; 7) southeast Tibet; 8) the Three Parallel Rivers areas; 9) the Xishuangbanna; 10) the Greater Khingan area; 11) the Sanjiang Plain; 12) the northern Bohai Rim, 13) the central Yangtze Plain; 14) the Yangtze River Delta; 15) the southeastern coastline area; 16) the Hainan Island and 17) the Taiwan Island.

Existing NNRs on the Qinghai-Tibet Plateau and the Qinling Mountains had well covered regions 5) the eastern Qilian Mountains and 7) the Qinling Mountains in Fig 5A under RCP 2.6, regions 4) the western Kunlun Mountains, 5) the Qilian Mountains, and 6) eastern Sanjiangyuan area in Fig 5B under RCP 8.5, providing potentials for species adaption to future climate changes. However, the overall coverages of the top 30% of priority areas based on NNRs are low at only 8.01% for RCP 2.6 and 12.03% for RCP 8.5.

In the meantime, we ranked priorities outside of existing NNRs using NNRs masked scenario in Fig 5C and 5D. Clearly, conservation gaps are significant. For RCP 2.6 (Fig 5C), top priorities that need to be conserves would be: 1) the Altai Mountains; 2) the Tianshan Mountains; 3) the Karakoram Mountains; 4) the southeastern Tibet; 5) the area cross borders of Qinghai, Gansu, and Sichuan provinces; 6) central Yunnan mountains; 7) south Yunnan; 8) the Greater Khingan area; 9) the Bohai Rim; 10) the central Yangtze River Basin; 11) the Yangtze River Delta and the Hangzhou Bay; 12) southeastern coastline areas; 13) the Hainan Island; and 14) the Taiwan Island. For RCP 8.5 (Fig 5D), top priorities out of NNRs are 1) the Altai Mountains; 2) the Karakoram Mountains; 3) southeast Tibet; 4) eastern Sanjiangyuan area; 5) the Three Parallel Rivers areas; 6) the Xishuangbanna; 7) the Greater Khingan area; 8) the Sanjiang Plain; 9) the Bohai Rim, 10) the central Yangtze Plain; 11) the Yangtze River Delta; 12) the southeastern coastline area; 13) the Hainan Island and 14) the Taiwan Island.

Furthermore, the Zero Draft suggested that 50% of the land area should be included in conservation planning, which require more priority areas should be conserved (shown in red, orange, yellow and light blue in Fig 5). Regardless of which emission scenario is considered, the entire eastern plain region of China, the eastern part of the Qinghai-Tibet Plateau, the Southwestern Mountains, the northern part of the Northeast China and the mountains of Xinjiang, should be priority areas for conservation. These areas mainly overlap with China's densely populated areas, especially in the eastern region.

## 4. Discussion

### 4.1 Citizen science plays an important role in assessing China’s bird distribution and its correspondence to climate change

China is a bird-rich country where the land area spans four of the nine major migration routes (the EAAF, the Asian–East African Flyway, the Central Asian Flyway and the West Pacific Flyway) in the world and contains 77 endemic bird species. For example, the EAAF, which is used by many threatened species and shared by 22 countries from Russia to New Zealand, and 246 bird species migrate through China [48]. Assessing the distribution of Chinese birds and their corresponding responses to climate change is indispensable for international bird conservation planning. This is made possible by the availability of a large-scaled citizen science dataset. The users can download richness maps for the current, RCP 2.6, and RCP 8.5 scenarios as geoTIFFs (S3 File).

In past two decades, an increasing number of citizens in China have become interested in birdwatching and shared their data in an open-source manner. This study, benefited from this citizen science data, may serve as a baseline for site-based conservation planning, ecosystem service accounting, and ecological process research. Currently the Bird Report is the only open source bird data with accurate species occurrence points available in China as each species record is verified by an experienced reviewer to minimize errors in species identification and locality.

Despite the potential sampling bias, citizen science can provide a wide range of occurrence data in a short time, providing a basis for rapid assessment. Species distribution model-based conservation and decision-making using citizen science data has been widely accepted by researchers as evidence-based approaches for conservation planning [71]. It is true that, in SDMs such as MaxEnt, different parameter setting approaches may lead to different output results [72] and it is necessary to correct sampling bias. In this study, we used the presence thinning and background selection methods for sampling bias correction [46, 47, 61]. For variable selection in this study, we removed highly correlated and low contribution variables to avoid overfitting [64]. However, we found that some island (the Taiwan Island and the Hainan Island) and endemic species (18 island species have a 0.26 training AUC reduction on average) had poor performance perhaps due to their small sampling background (island areas) [73]. This suggests that more species-specific modeling methods should be applied for species with special distribution pattern. We believe that according to different research goals, comprehensive consideration of various correction methods can maximize the advantages of citizen scientific data in distribution modelling, not to say the low economic cost for data collection.

### 4.2 Uncertainty of bird survival in China under climate change

Our results reveal that the impact of climate change on the distribution of birds is significant in China, both reducing or expanding in the range area and shifting in its elevation or latitude. In general, our assessment indicated that: 1) more birds would have opportunities to expand their ranges than would face risks related to habitat loss in China due to climate change in the next 50 years, similar to the report on a national-scale assessment of more than 3,000 species in the U.K. [74]; 2) most bird species may move to northeast or northwest China and high altitude areas (this trend is similar to the review results summarized by Davis and Shaw, 2001 and Pearce-Higgins et al., 2011 [53, 74]). 3) compared with the range change of resident birds, the range area of migratory species is more likely to move northward and decrease in area, which suggests that migratory birds may be more threatened by future climate change.

Special attention should be paid to the 25% of the birds that may reduce the range size. For example, Red-crowned Crane (*G*. *japonensis*), a popular endangered species which has lost 92% wintering range in past 30 years [75], may face the risk of losing 94% of its existing range area in the next 50 years (S2 Fig). The current conservation strategy on the species recovery may not be adequate to deal with the threat of future climate change. Close monitoring to the population and investigation to its potential future habitats is needed [76].

For the 75% of the birds that may expand the range size, however, the situation is not necessarily positive either if their future climatic suitable habitats are away from protected areas or toward more developed land. With the shift and expansion of the distribution range, bird species may enter a climatically suitable, but high-intensity populated habitats, and thus face the threat of poaching and illegal trade, urbanization, and industrialization. For example, all of the top ten “expanders” under RCP 8.5 may move to developed and densely populated areas, such as the Pearl River Delta and surrounding Beijing suburbs (S2 Fig).

### 4.3 A more rigorous conservation system is needed for China

While species range shifting, the conservation priority areas also shift with the future climate influence. We identified the future conservation priority areas for birds in China using top 30% threshold to meet the target of land conservation proposed in Zero Draft of the Post-2020 Global Biodiversity Framework [77]. The national nature reserves only cover 6.73% of priority areas for RCP 2.6 and 9.9% for RCP 8.5, which is inadequate to cope with the climate impact. Many important habitats remain as conservation gaps. For example, many critical coastal wetlands are not listed as nature reserves [36], where many severely affected bird species may move to (S2 Fig). More protection is also needed in in the Northeast Plain, Bohai Rim/North China Plain, Karakoram Mountain, Lower Yangtze Basin, Southwestern Mountains and northern Xinjiang areas. Policy-makers must integrate these research results combined from different taxa so that future NNR systems in China can mitigate the effects of climate change [18].

However, an obvious characteristic of the priorities we identified in eastern China is intensive human development. Establishing official and sizable protected areas may become difficult. It is necessary to consider the restoration of species habitats and connectivity even in urban parks and greening zones, as well as land-sharing strategies to accommodate biodiversity in farmlands [39], in order to reach the more ambitious conservation targets. A new “inclusive” conservation model, including an eco-friendly lifestyle, would be an eventual solution. Citizen awareness and choices will be critical in this approach, and indigenous knowledge may result in high value being given to preserving biodiversity [34].

In this study, a comprehensive analysis based on point data of bird distributions is performed; notably, this analysis is the first of its kind in China. Additionally, our bird data were only collected in China, which limited our analysis to potential changes in bird movements across the boundaries of China, especially from Southeast Asia and Central Asia. Moreover, with the expansion of bird watching and the increase in data size and quality, we hope that periodic assessments such as the analysis in this study will be continuously updated to address the challenges associated with rapid changes in biodiversity, and to provide available distribution data for government agencies and researchers.

## Supporting information

S1 Fig Richness maps for each RCP based on breeding & resident ranges. A-C are the breeding range pattern of 211 migratory species (A: current, B: RCP 2.6, C: RCP 8.5); D-F are the richness pattern of “breeding range of migratory species plus range of resident species” group (D: current, E: RCP 2.6, F: RCP 8.5); G-H show the richness change under RCP 2.6 (G) and RCP 8.5 (H). The boundaries are reprinted from Shan Shui Conservation Center under a CC BY 4.0 license, with permission from Xiangying Shi, original copyright 2020.
(TIF)

S2 Fig Range change maps for the top 10 species with the highest range loss/expansion for each RCP. The boundaries are reprinted from Shan Shui Conservation Center under a CC BY 4.0 license, with permission from Xiangying Shi, original copyright 2020.
(TIF)

S3 Fig Richness maps for each RCP. The boundaries are reprinted from Shan Shui Conservation Center under a CC BY 4.0 license, with permission from Xiangying Shi, original copyright 2020.
(TIF)

S1 Table Checklist, number of localities, distribution range, MaxEnt results, latitude shift data, and weight value used in Zonation of study bird species. (XLSX)

S2 Table Checklist, number of localities, distribution range, and MaxEnt results of breeding range modelling. (XLSX)

S1 Video 3D model. 3D scene models of the vertical structure changes of the richness pattern for different RCPs. The model is shown as a mp4 video file.
(MP4)

S1 File Additional methods and results. Methods and results for modelling breeding range of migratory species.
(DOCX)

S2 File Script. S2-1: The R script for background selection and variable selection. S2-2: The Python script for batch run MaxEnt model.
(DOCX)

S3 File Raster. Original richness map data in geoTIFFs.
(RAR)
