# Supplementary figures and images for: Shifts in bird ranges and conservation priorities in China under climate change
Source: PLoS One. 2020 Oct 8;15(10):e0240225. doi: 10.1371/journal.pone.0240225 (PMC7544134; doi:10.1371/journal.pone.0240225)

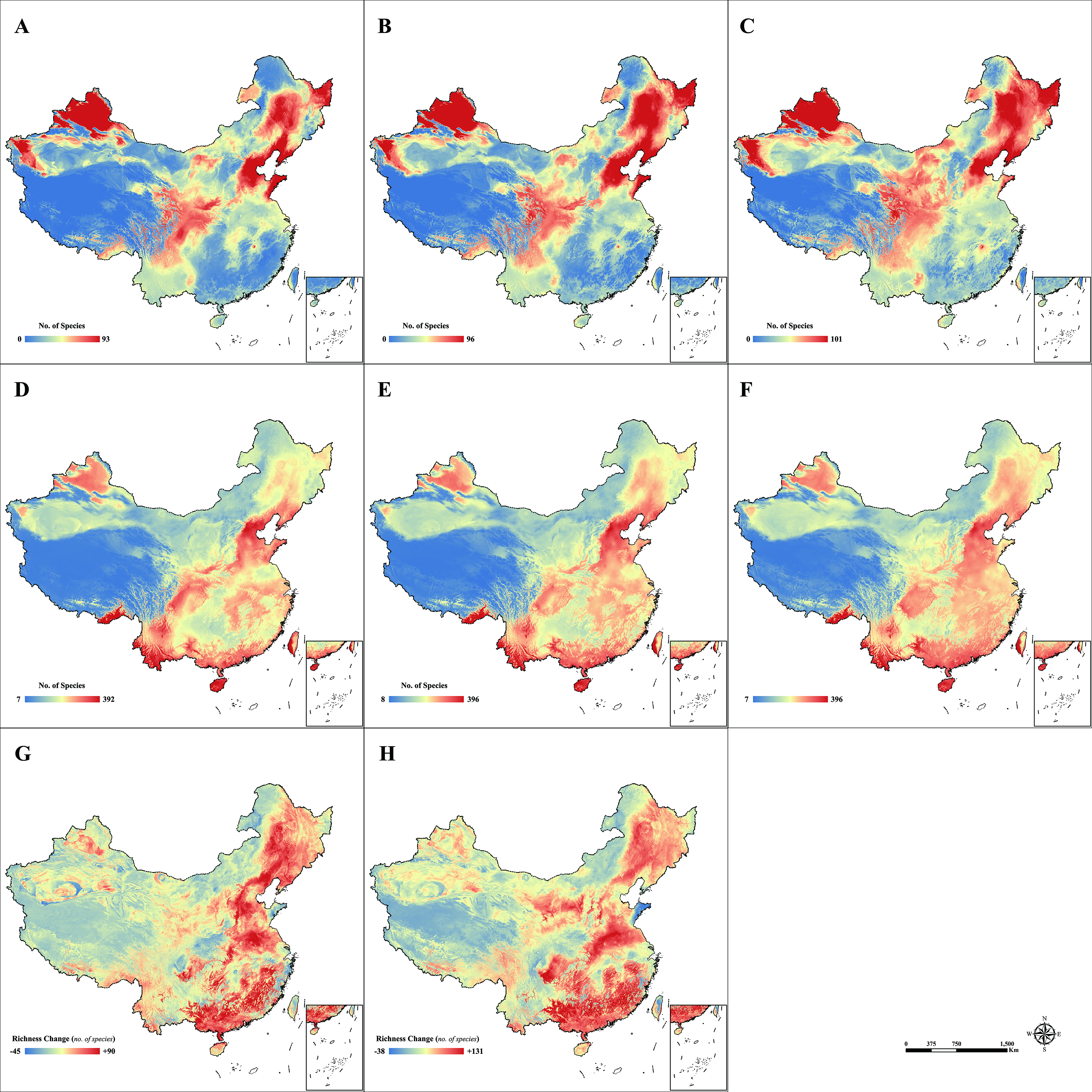

Supplement: S1 Fig — A-C are the breeding range pattern of 211 migratory species (A: current, B: RCP 2.6, C: RCP 8.5); D-F are the richness pattern of “breeding range of migratory species plus range of resident species” group (D: current, E: RCP 2.6, F: RCP 8.5); G-H show the richness change under RCP 2.6 (G) and RCP 8.5 (H). The boundaries are reprinted from Shan Shui Conservation Center under a CC BY 4.0 license, with permission from Xiangying Shi, original copyright 2020. (TIF) [file pone.0240225.s001.tif]

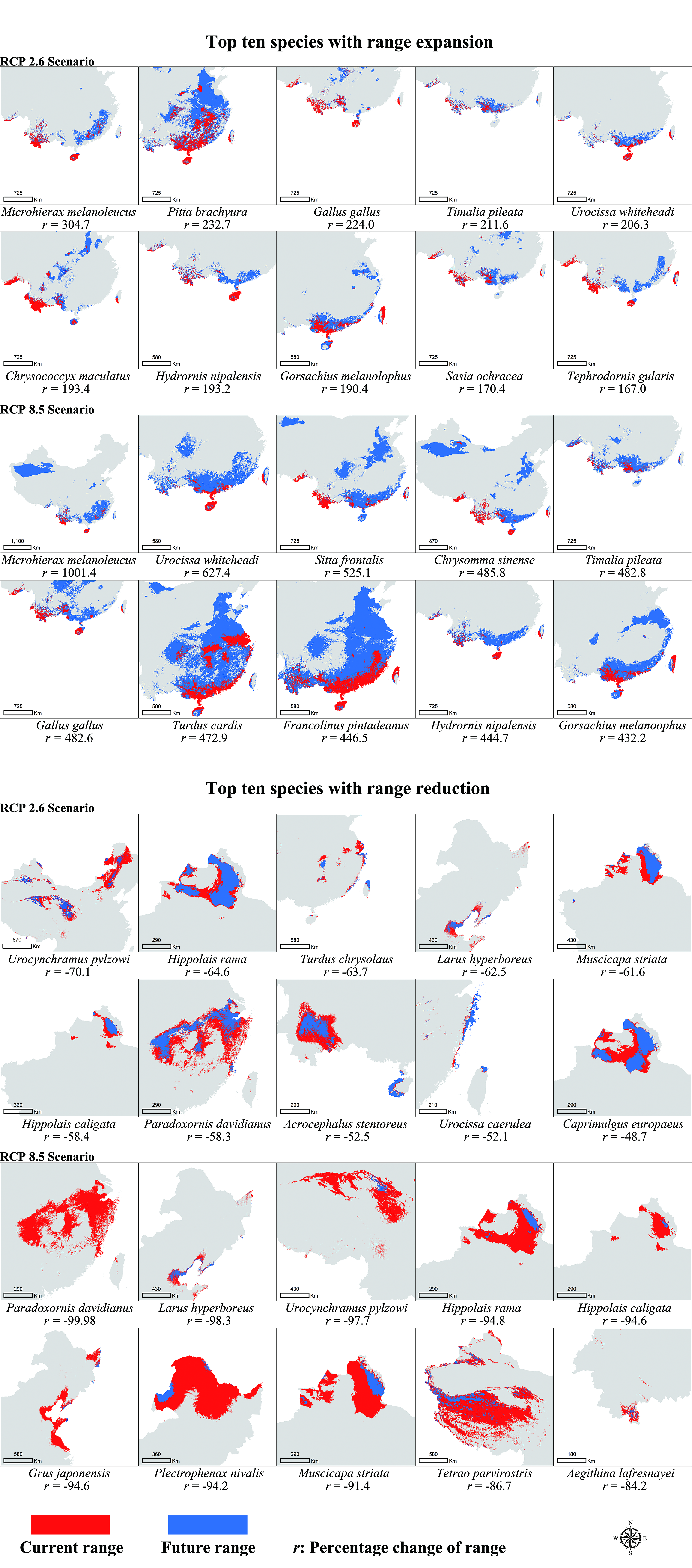

Supplement: S2 Fig — The boundaries are reprinted from Shan Shui Conservation Center under a CC BY 4.0 license, with permission from Xiangying Shi, original copyright 2020. (TIF) [file pone.0240225.s002.tif]

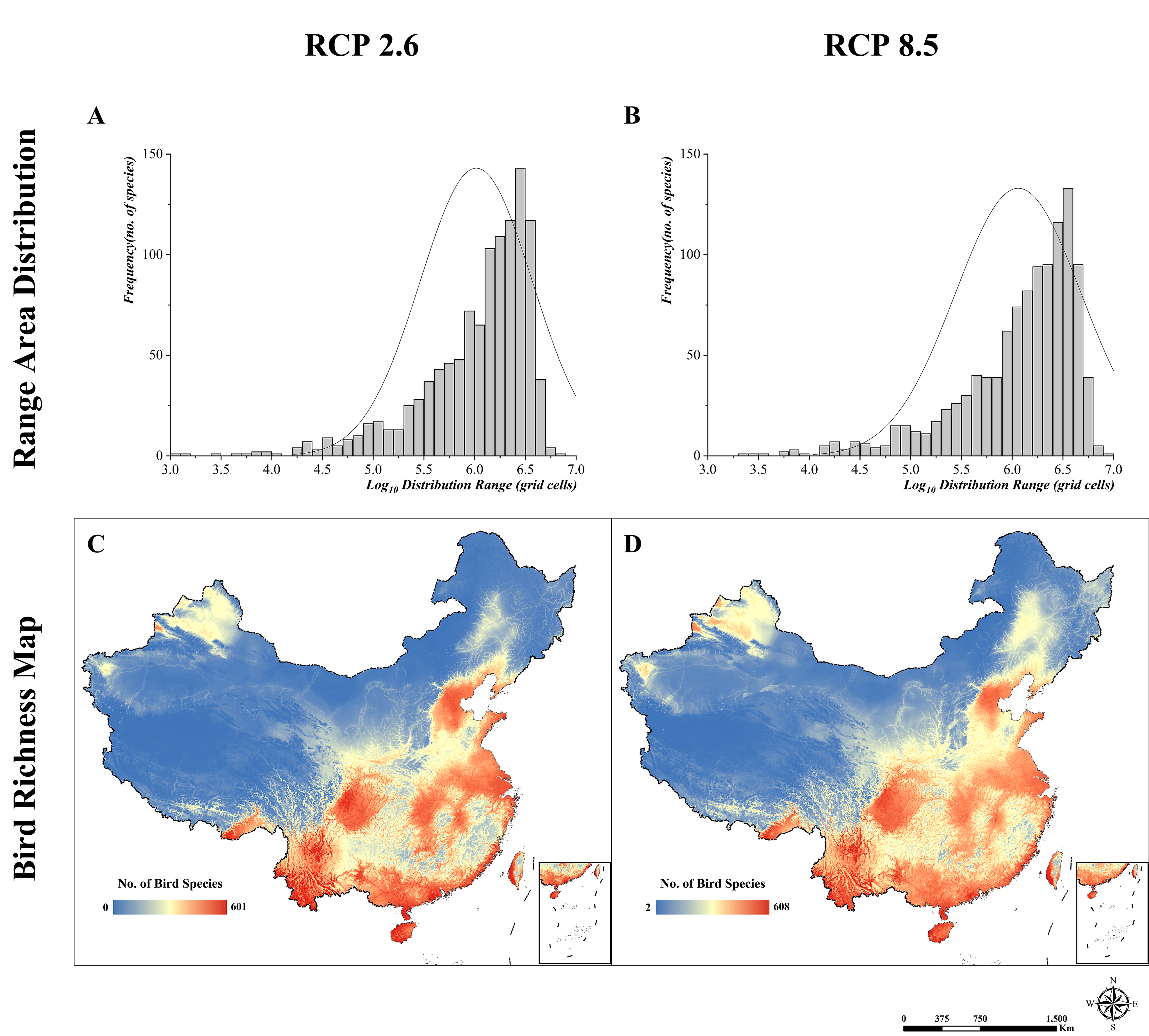

Supplement: S3 Fig — The boundaries are reprinted from Shan Shui Conservation Center under a CC BY 4.0 license, with permission from Xiangying Shi, original copyright 2020. (TIF) [file pone.0240225.s003.tif]
