# Supplementary material for: Shifts in bird ranges and conservation priorities in China under climate change
Source: PLoS One. 2020 Oct 8;15(10):e0240225. doi: 10.1371/journal.pone.0240225 (PMC7544134; doi:10.1371/journal.pone.0240225)
Supplement: S1 File — Methods and results for modelling breeding range of migratory species. (DOCX) [file pone.0240225.s007.docx]

**Methods for mapping breeding range of migratory species**

For each of 565 migratory bird species in first-round process, we obtained the breed occurrence data by filtering by the IUCN breeding extant map in China. After filtering, we found that: 1) 271 species have no breeding ranges (traveling or wintering birds) in China; 2) 22 species have the same number of distribution points before and after filtering (all distribution points are in the breeding range); 3) 52 species have less than five remaining points after filtering; 4) 30 species have low locality density (so MaxEnt model cannot be used). Finally, the breeding range of other 190 migratory species with 12,966 independent breeding localities need to be mapped. We used the same variables selection, background selection, and parameter setting as the section 2.1 & 2.2 in the main text for modeling the presence/absence breeding range maps.

**Results**

**Breeding range modelling**

After filtering, we found that on average, the percentage of breeding localities in total is 43.1%. The mean training AUC value of these species is 0.942±0.029; the mean test AUC value is 0.907±0.047 (See Supporting information Table 1).

**Range area change under different scenarios**

The mean percentage of breeding range in total range for the current scenario, RCP 2.6 scenario, and RCP 8.5 scenario is 74.32%, 67.09%, and 64.67%, respectively. Seventy-three species may lose 14.78% breeding ranges, and the other 117 species may have a 28.58% breeding range expansion on average under RCP 2.6. For RCP 8.5, there are 66 species may suffer a 33.62% breeding range loss and 124 species may have a 69.52% breeding range expansion on average (See Supporting information Table S2). In general, for these species, future climate change may have less impact on their breeding range area than on total distribution range areas. In other words, the breeding range will be relatively stable under climate change.

**Richness pattern change under different scenarios**

We overlaid the range map for each species to show the species richness pattern. The breeding richness pattern of 190 migratory species (Figure S4 A-C) is different from their total richness pattern (Figure 4B), for the hotspot of breeding richness pattern is consists of the Northeast Plain, the north Xinjiang, and the Bohai Rim. Regardless of the climate scenarios, the richness pattern of “grand resident” group (Figure S4 D-F), consist of 190 breeding species, 22 original species and 516 resident species, is also different from the total pattern described in the main document (Figure 4A, Supporting information S3 Figure C&D), mainly manifested in the distribution of the high-diversity area is further south. These results show that if we no longer consider wintering and migrant ranges, the understanding of patterns and the critical regions for conservation will be very different.
