# Supplementary material for: Shifts in bird ranges and conservation priorities in China under climate change
Source: PLoS One. 2020 Oct 8;15(10):e0240225. doi: 10.1371/journal.pone.0240225 (PMC7544134; doi:10.1371/journal.pone.0240225)
Supplement: S2 File — S2-1: The R script for background selection and variable selection. S2-2: The Python script for batch run MaxEnt model. (DOCX) [file pone.0240225.s008.docx]

**S2-1 R script for background selection and variable selection:**

library(raster)

myDF = read.csv("birds_prov&var.csv")

bgDir = "bg"

myRas = raster("province2.tif")

xmin=extent(myRas)[1]

xmax=extent(myRas)[2]

ymin=extent(myRas)[3]

ymax=extent(myRas)[4]

step=1/120

ny = nrow(myRas)

nx = ncol(myRas)

myRasDF = data.frame(prov=values(myRas),

long=rep(seq(xmin+0.5*step, xmax-0.5*step, step),times=ny),

lat=rep(seq(ymax-0.5*step, ymin+0.5*step, -step),each=nx))

myRasDF = subset(myRasDF,(prov>=1)&(prov<=34))

nbg = 10000

for (i in 1:1042){

print(paste0(i,",",as.character(myDF[i,"Species"])))

provlist = unlist(strsplit(as.character(myDF[i,"Province"]),','))

mySubDF = subset(myRasDF, prov %in% provlist)

write.csv(mySubDF[sample(1:nrow(mySubDF),nbg),],

paste0(bgDir, "\\", myDF[i,"Species"],"_bg.csv"),row.names=FALSE)

}

myDF = read.csv("birds_prov&var.csv")

envDir = "F:\\Bird\\proj\\current"

bgDir = "bg"

presDir = "csv"

outDir = "swd"

for (i in 1:1042){

print(paste0(i,",",as.character(myDF[i,"Species"])))

spID = as.character(myDF[i,"Species"])

varlist = unlist(strsplit(as.character(myDF[i,"Variable"]),','))

envfiles = paste0(envDir, "\\", varlist, ".asc")

myEnvStack = stack(envfiles)

myPres = read.csv(paste0(presDir, "\\", spID, ".csv"))

colnames(myPres) = c("species","long","lat")

myPres$species = spID

myBg = read.csv(paste0(bgDir, "\\", spID, "_bg.csv"))

myBg$species = ""

myPt = rbind(myPres[,c(1,2,3)],myBg[,c(4,2,3)])

mySwd = cbind(myPt,extract(myEnvStack,myPt[,2:3]))

write.csv(na.omit(mySwd),paste0(outDir, "\\", spID, "_swd.csv"),row.names=FALSE)

}

**S2-2 Python script for MaxEnt modeling:**

# -*- coding: UTF-8 -*-

# write by Mei Luo

# --PACKAGE--

import os, time, pandas as pd

import arcpy, dbfread

# --INPUT/OUTPUT--

workDir = "F:\\Bird"

inStatFile = "birds_prov&var.csv"

outStatFile = "birds_stat1.csv"

inDir = "F:\\Bird\\swd"

outDir1 = "F:\\Bird\\maxentoutput"

outDir2 = "F:\\Bird\\output"

projdir = "%s\\current,%s\\cc2_6,%s\\cc8_5,%s\\he2_6,%s\\he8_5,%s\\mc2_6,%s\\mc8_5"%(tuple(["F:\\Bird\\proj"])*7)

maxentFile = "G:\\new_maxent\\maxent.jar"

thrField = "Maximum test sensitivity plus specificity Cloglog threshold"

# --PREPROCESS--

os.chdir(workDir)

if (not os.path.exists(outDir1)): os.makedirs(outDir1)

if (not os.path.exists(outDir2)): os.makedirs(outDir2)

arcpy.CheckOutExtension("Spatial")

arcpy.env.overwriteOutput = True

arcpy.env.pyramid = "NONE"

arcpy.env.workspace = workDir

# --FUNCTION--

def RunMaxent(spfile, envdir, outputdir, projdir = projdir, maxent = maxentFile, logfile = "maxent.log"):

runPresentMinimal = "java -Xmx6g -jar " + maxent + " responsecurves=false jackknife=false outputgrids=false" + \

" outputdirectory=" + outputdir + \

" projectionlayers=" + projdir + \

" samplesfile=" + spfile + \

" environmentallayers=" + envdir + \

" replicates=5 replicatetype=crossvalidate" + \

" warnings=false askoverwrite=false skipifexists=false autorun=true threads=4" + \

" logfile=" + logfile

os.popen(runPresentMinimal)

# --MAIN--

inStatDF = pd.read_csv(inStatFile)

outStatDF = pd.DataFrame()

for i in range(0, 1041):

spID = inStatDF.ix[i,"Species"]

print time.strftime("%Y-%m-%d %H:%M:%S Start:", time.localtime()), i, spID

mxntDir = os.path.join(outDir1, spID)

if (not os.path.exists(mxntDir)): os.makedirs(mxntDir)

RunMaxent(os.path.join(inDir, spID+"_swd.csv"),

os.path.join(inDir, spID+"_swd.csv"),

mxntDir)

result_csv = pd.read_csv(os.path.join(mxntDir, "maxentResults.csv"))

#outStatDF = outStatDF.append(result_csv.ix[5],ignore_index=True)

thr = result_csv.ix[len(result_csv)-1, "Maximum test sensitivity plus specificity Cloglog threshold"]

outStatDF = outStatDF.append(inStatDF.ix[i])

outStatDF.ix[i, 'Threshold MTSS'] = result_csv.ix[len(result_csv)-1, "Maximum test sensitivity plus specificity Cloglog threshold"]

outStatDF.ix[i, 'Training AUC'] = result_csv.ix[len(result_csv)-1, 'Training AUC']

outStatDF.ix[i, 'Test AUC'] = result_csv.ix[len(result_csv)-1, 'Test AUC']

outStatDF.ix[i, 'AUC Standard Deviation'] = result_csv.ix[len(result_csv)-1, 'AUC Standard Deviation']

current_asc = arcpy.Raster(os.path.join(mxntDir, spID+"_current_avg.asc"))

rcp26_asc = (arcpy.Raster(os.path.join(mxntDir, spID+"_cc2_6_avg.asc")) + \

arcpy.Raster(os.path.join(mxntDir, spID+"_he2_6_avg.asc")) + \

arcpy.Raster(os.path.join(mxntDir, spID+"_mc2_6_avg.asc")) )/3

rcp85_asc = (arcpy.Raster(os.path.join(mxntDir, spID+"_cc8_5_avg.asc")) + \

arcpy.Raster(os.path.join(mxntDir, spID+"_he8_5_avg.asc")) + \

arcpy.Raster(os.path.join(mxntDir, spID+"_mc8_5_avg.asc")) )/3

current_asc.save(os.path.join(outDir2, spID + "_current.tif"))

rcp26_asc.save(os.path.join(outDir2, spID + "_rcp26.tif"))

rcp85_asc.save(os.path.join(outDir2, spID + "_rcp85.tif"))

remap = arcpy.sa.RemapRange([[0, thr, 0],[thr, 1, 1]])

arcpy.sa.Reclassify(os.path.join(outDir2, spID + "_current.tif"), "Value", remap).save(os.path.join(outDir2, spID + "_current_01.tif"))

arcpy.sa.Reclassify(os.path.join(outDir2, spID + "_rcp26.tif"), "Value", remap).save(os.path.join(outDir2, spID + "_rcp26_01.tif"))

arcpy.sa.Reclassify(os.path.join(outDir2, spID + "_rcp85.tif"), "Value", remap).save(os.path.join(outDir2, spID + "_rcp85_01.tif"))

myDbf = dbfread.DBF(os.path.join(outDir2, spID+"_current_01.tif.vat.dbf"))

for record in myDbf:

if (record['VALUE']==1):

outStatDF.ix[i, 'No.Cells_current'] = record['COUNT']

myDbf = dbfread.DBF(os.path.join(outDir2, spID+"_rcp26_01.tif.vat.dbf"))

for record in myDbf:

if (record['VALUE']==1):

outStatDF.ix[i, 'No.Cells_rcp26'] = record['COUNT']

myDbf = dbfread.DBF(os.path.join(outDir2, spID+"_rcp85_01.tif.vat.dbf"))

for record in myDbf:

if (record['VALUE']==1):

outStatDF.ix[i, 'No.Cells_rcp85'] = record['COUNT']

arcpy.sa.ZonalGeometryAsTable(os.path.join(outDir2, spID + "_current_01.tif"), "Value", os.path.join(outDir2, spID+"_current_01.dbf"))

arcpy.sa.ZonalGeometryAsTable(os.path.join(outDir2, spID+"_rcp26_01.tif"), "Value", os.path.join(outDir2, spID+"_rcp26_01.dbf"))

arcpy.sa.ZonalGeometryAsTable(os.path.join(outDir2, spID+"_rcp85_01.tif"), "Value", os.path.join(outDir2, spID+"_rcp85_01.dbf"))

myDbf = dbfread.DBF(os.path.join(outDir2, spID+"_current_01.dbf"))

for record in myDbf:

if (record['VALUE']==1):

outStatDF.ix[i, 'Area_current'] = record['AREA']

outStatDF.ix[i, 'X Centroid_current'] = record['XCENTROID']

outStatDF.ix[i, 'Y Centroid_current'] = record['YCENTROID']

myDbf = dbfread.DBF(os.path.join(outDir2, spID+"_rcp26_01.dbf"))

for record in myDbf:

if (record['VALUE']==1):

outStatDF.ix[i, 'Area_rcp26'] = record['AREA']

outStatDF.ix[i, 'X Centroid_rcp26'] = record['XCENTROID']

outStatDF.ix[i, 'Y Centroid_rcp26'] = record['YCENTROID']

myDbf = dbfread.DBF(os.path.join(outDir2, spID+"_rcp85_01.dbf"))

for record in myDbf:

if (record['VALUE']==1):

outStatDF.ix[i, 'Area_rcp85'] = record['AREA']

outStatDF.ix[i, 'X Centroid_rcp85'] = record['XCENTROID']

outStatDF.ix[i, 'Y Centroid_rcp85'] = record['YCENTROID']

outStatDF.to_csv(outStatFile)

# END of for spID
